# The Prebiotic Potential of Geraniin and Geraniin-Enriched Extract against High-Fat-Diet-Induced Metabolic Syndrome in Sprague Dawley Rats

**DOI:** 10.3390/antiox11040632

**Published:** 2022-03-25

**Authors:** Mohanambal Moorthy, Chong Chun Wie, Eliana Mariño, Uma D. Palanisamy

**Affiliations:** 1 Jeffrey Cheah School of Medicine and Health Sciences, Monash University Malaysia, Jalan Lagoon Selatan, Bandar Sunway 47500, Selangor, Malaysia; mohanambal.moorthy@monash.edu; 2 School of Pharmacy, Monash University Malaysia, Jalan Lagoon Selatan, Bandar Sunway 47500, Selangor, Malaysia; chong.chunwie@monash.edu; 3 Monash Biomedicine Discovery Institute, Monash University Clayton Campus, 19 Innovation Walk, Clayton, VIC 3800, Australia; eliana.marino@monash.edu

**Keywords:** *Nephelium lappaceum* L., ellagitannin, metabolic syndrome, gut microbiota, prebiotic

## Abstract

Geraniin, an ellagitannin, has ameliorative properties against high-fat diet (HFD)-induced metabolic syndrome. Since geraniin has poor bioavailability, we hypothesised the interaction of this compound with gut microbiota as the main mechanism for improving metabolic aberrations. Male Sprague Dawley rats were divided into normal diet (ND)- and HFD-fed animals and treated with geraniin and an enriched extract of geraniin (GEE). We observed that 5 mg geraniin and 115 mg GEE supplementation significantly attenuated glucose intolerance, lipopolysaccharide-binding protein, total cholesterol, triacylglyceride, and low-density lipoprotein; improved insulin sensitivity; and significantly increased adiponectin and hepatic PPARα expression. Although geraniin and GEE did not significantly alter the gut microbial composition, we found an increment in the relative abundance of a few butyrate producers such as *Alloprevotella*, *Blautia*, *Lachnospiraceae NK4A136 group*, and *Clostridium sensu stricto 1*. Geraniin and its enriched extract’s ability to ameliorate metabolic syndrome parameters while positively affecting the growth of butyrate-producing bacteria suggests its potential prebiotic role.

## 1. Introduction

Metabolic syndrome (MetS) comprises a cluster of metabolic abnormalities, including central obesity, high triacylglyceride (TG), high blood pressure, high fasting blood glucose, and low high-density lipoprotein [1]. The condition is diagnosed by the concomitant presence of at least three of the five abovementioned metabolic abnormalities, increasing the risk of developing various diseases, particularly cardiovascular disease [2] and type-II diabetes [3]. The pathogenesis of MetS is mainly associated with oxidative stress (OS).

Exposure to excess energy causes cell death and releases chemokines that attract monocytes and macrophages (M2) [4,5,6]. The increased M2 macrophages also generate ROS and release a variety of proinflammatory cytokines. Proinflammatory cytokines such as TNF have been shown to induce insulin resistance (IR) in adipocytes, which in turn augments very-low-density lipoprotein (VLDL) synthesis, decreases lipoprotein lipase, and increases TG, exacerbating obesity-related OS [4,5,6].

The global prevalence of metabolic syndrome in 2006 was estimated as 20 to 25%, affecting 1.6 billion of the world’s population [7], which had increased to approximately 2 billion by 2018 [8]. This growing incidence indicates that existing strategies such as lifestyle modifications are insufficient, mainly due to patients’ lack of motivation [9]. However, pharmacotherapies are expensive and associated with side effects [10]. Alternatively, manipulation of gut microbiota seems to be a promising approach in managing metabolic syndrome.

Gut microbiota profoundly influence our health due to their role in immunity, vitamin synthesis, satiety regulation, and energy harvest [11]. Hence, gut dysbiosis, i.e., imbalance of the healthy gut microbiota, has been associated with obesity and metabolic syndrome [11]. Alterations of the gut microbiota induced by a HFD are strongly linked to weight gain, adiposity, glucose intolerance, insulin resistance, inflammation, and oxidative stress [12]. Subsequent studies have utilised the prebiotic nature of plant-based compounds to improve gut microbiota composition and attenuate HFD-inflicted metabolic derangements [13,14].

Polyphenols are compounds found abundantly in fruits and vegetables that promote health [15]. However, polyphenols have poor bioavailability due to their structural complexity and polymerisation, interaction with the food matrix, and easy degradation by an acid or alkaline environment [16]. The gut microbiota play a critical role in converting these phytochemicals into bioactive metabolites that affect the intestinal ecology, gut homeostasis, and overall host health [15]. Ellagitannins, the less explored polyphenol, have been shown to have promising health outcomes in association with their ability to positively reshape GM composition [13,14].

Geraniin, an ellagitannin found predominantly in Geranium, *Euphorbia*, and *Phyllanthus* species [17], was also discovered in the rind of *Nephelium lappaceum* L. (commonly known as rambutan) [18]. The rind is a rich source of ellagitannins such as geraniin and corilagin [18], with geraniin being the primary compound [18,19]. In a diet-induced animal model, varying doses of geraniin were reported to attenuate hyperglycaemia, dyslipidaemia, and hypertension [20,21,22]. As geraniin has poor bioavailability [23], we hypothesised that the interaction of geraniin with gut microbiota and the resulting metabolites are together responsible for ameliorating metabolic derangements induced by the high-fat diet. Although the bioactivity of GEE is comparable to geraniin [19,24], the efficacy of GEE against diet-induced metabolic syndrome has never been investigated. Hence, this study examines the effect of geraniin and its enriched extract (GEE) on gut microbiota composition, metabolic endotoxemia, cytokines, gene expression (inflammation and lipid metabolism), short-chain fatty acids, and metabolic parameters in a diet-induced metabolic syndrome animal model. Additionally, the metabolic effects of geraniin and its enriched extract under normal physiological conditions were evaluated among the normal-diet-fed rats.

## 2. Materials and Methods

### 2.1. Preparation of Geraniin Enriched Extract (GEE) and Geraniin

*N. lappaceum* L. fruits were purchased in Kuala Lumpur, Malaysia, which were authenticated by the Herbarium of the Forest Research Institute of Malaysia (FRIM). The rinds of the fruits were used in this study. The extraction of GEE and the purification process to produce geraniin crystals have been published elsewhere [25]. The constituents of GEE and the identity of geraniin were confirmed using liquid chromatography-mass spectroscopy (LCMS).

### 2.2. Animal Study

This animal study was approved by the Monash University Monash Animal Research Platform Animal Ethics Committees (AEC approval no.: MARP/2017/01). Three-week-old male Sprague Dawley (SD) rats were purchased from the Monash Animal Research Platform. The rats were acclimatised under controlled conditions of humidity with a 12 h light/dark cycle, with ad libitum access to food and water, for one week. The rats were then randomly divided into seven groups (*n* = 8/group): normal diet (ND), normal diet supplemented with 50 mg/kg bw geraniin (ND + 50GE), normal diet supplemented with 115 mg/kg bw of geraniin-enriched extract (ND + GEE), high-fat diet (HFD), HFD supplemented with 5 mg/kg bw of geraniin (HFD + 5GE), HFD supplemented with 50 mg/kg bw of geraniin (HFD + 50GE), and HFD supplemented with 115 mg/kg bw of geraniin-enriched extract (HFD + GEE). The rats were fed a normal diet (AIN93G D10012GR, Research Diets) or an HFD (AIN93G D12033001, Research Diets) for 12 weeks. The composition and energy densities of the experimental diets are presented in Supplementary Table S1. From the ninth week onwards, the control groups (ND, HFD) were gavaged with distilled water, and the remaining groups were gavaged with geraniin or GEE once a day for four weeks. During the entire study period of 12 weeks, food and body weight were assessed weekly.

### 2.3. Sample Collection

Faeces were collected at week 13, immediately snap-frozen, and stored at −80 °C. At the endpoint of the experiment, the rats were fasted for 12 h and were anaesthetised by intraperitoneal injection of ketamine (100 mg/kg) and xylazine (10 mg/kg). Cardiac puncture was performed to collect blood, and plasma was separated and stored at −80 °C until further analysis. Liver and white adipose tissues were removed, snap-frozen immediately, and stored at −80 °C.

### 2.4. Lipid Profile

Plasma samples were sent to the Haematology Unit, Veterinary Faculty, Universiti Putra Malaysia, for measurement of total cholesterol (TC), triacylglyceride (TG), high-density lipoprotein (HDL), and low-density lipoprotein (LDL).

### 2.5. Glucose Homeostasis

At the end of the 12th week, an oral glucose tolerance test (OGTT) was performed on 12 h fasted SD rats using a glucose meter (Accu Check^®^ Performa). Blood glucose concentration was determined before (0 min) and after (15, 30, 60, 90, 120 min) oral glucose administration. The fasting insulin (FI) level was measured using the Mercodia Ultrasensitive Rat Insulin ELISA (Mercodia, Sweden). The homeostatic model assessment of insulin resistance index (HOMA IR) was calculated using a HOMA IR calculator https://www.thebloodcode.com/homa-ir-calculator/ (accessed on 7 June 2019).

### 2.6. Plasma Lipopolysaccharide-Binding Protein

The lipopolysaccharide-binding protein (LBP) concentration in plasma was measured using the LBP ELISA kit (Cloud Clone Corp.) according to the manufacturer’s instructions.

### 2.7. Gene Expression

Total RNA was extracted from homogenised liver and retroperitoneal white adipose tissues (rWAT) using a Macherey Nagel NucleoSpin RNA Plus kit and a QIAGEN RNeasy Plus Universal Mini kit, respectively. Following RNA reverse transcription (High-Capacity cDNA Reverse Transcription Kit, Applied Biosystems, Thermo Fisher Scientific), quantitative real-time PCR (qPCR) was performed using PowerUp SYBR Green Master Mix (Applied Biosystems, Thermo Fisher Scientific). Target genes were TNFα, Il 6, Il 1β, and peroxisome proliferator-activated receptor alpha (PPARα) for liver tissues, and peroxisome proliferator-activated receptor gamma (PPARγ) for rWAT tissues. Glyceraldehyde 3 phosphate dehydrogenase (GAPDH) was used as the reference gene for adipose tissue while lactate dehydrogenase A (LDHA) was used for liver. Supplementary Table S2 outlines the accession number and forward and reverse primer sequences of all the genes.

### 2.8. Plasma Adipocytokines

The levels of tumour necrosis factor-alpha (TNFα), interleukin 6 (IL 6), interleukin 1β (IL1β), monocyte chemoattractant protein 1 (MCP1), leptin, and adiponectin were measured using the MIILIPLEX^®^ Map Adipocyte Panel Metabolism Assay RADPCMAG 82k 06 in the Luminex Analyser MAGPIX^®^.

### 2.9. SCFAs Quantification in Plasma

Plasma samples were processed as per the method published by De Baere et al. in 2013 [26]. Briefly, standard curves were established for acetate, butyrate, and propionate using HPLC 1200. Plasma samples (250 µL) were spiked with formic acid (internal standard). The spiked samples were vortexed and then equilibrated at room temperature, to which 25 µL of HCl was added and vortexed. Diethyl ether (1.25 mL) was added, and after gentle shaking for 20 min, 125 µL of NaOH was added to the supernatant. The mixture was centrifuged, following gentle shaking for 20 min. The resulting aqueous phase was separated and mixed with 25 µL of HCl, and finally 100 µL of the aliquot was injected into HPLC for analysis. The wavelength used was 210 nm.

### 2.10. DNA Extraction and 16 s rRNA Sequencing

Six samples from each of the five groups (ND, HFD, HFD + 5GE, HFD + 50GE, HFD + GEE) were selected for 16S NGS. The normal-diet treatment groups were excluded from this investigation since geraniin and GEE did not significantly affect the metabolic parameters. Total genomic DNA was extracted using a Qiagen QIAamp Powerfecal DNA extraction kit according to the manufacturer’s protocol. DNA quality was checked with a nanophotometer (IMPLEN) followed by agarose gel electrophoresis. The DNA extracts were used to amplify the V3 V4 hypervariable regions using the forward primer (5′TCGTCGGCAGCGTCAGATGTGTATAAGAGACAGCCTACGGGNGGCWGCAGTCGTCGGCAGCGTCAGATGTGTATAAGAGACAGCCTACGGGNGGCWGCAG 3′) and reverse primer (5′ GTCTCGTGGGCTCGGAGATGTGTATAAGAGACAGGACT ACHVGGGTATCTAATCC 3′). Indexed libraries were prepared according to the Illumina 16S library preparation protocol and sequenced on an IlluminaMiSeq sequencer platform (Illumina, San Diego, CA, USA) at the Monash University Malaysia Genomics Facility.

### 2.11. Bioinformatics

A total of 3,665,508 raw sequences were produced. The data were then quality-filtered and trimmed with DADA2. The taxonomy was assigned based on the RefSeq + RDP reference sequence for DADA2 [27]. The final dataset consisted of 974,313 sequences converted into an amplicon sequence variant (ASV) abundance table and processed using the Phyloseq R package [28]. ASVs with sequences < 100 and present in < 5% of the samples were removed. Alpha diversity was assessed with Pielou’s evenness, richness index, Shannon diversity, and Simpson diversity using the Vegan R [29], whereby statistical significance was tested using ANOVA (*p* < 0.05). The abundance table comprised central log-ratio (CLR) transformed before beta diversity comparison. The beta diversity between groups was inferred based on principal coordinate analysis (PCoA) and canonical analysis of principal coordinates (CAP). Permutational multivariate analysis of variance (PERMANOVA) with the adonis2 function was conducted to test whether the GM composition was significantly different between the control (ND, HD) and treated groups. The taxonomy plot and the proportional differences in phyla and genera were analysed using the Phyloseq (version 1.8.2) package in R (accession date: 14 November 2020). ANOVA-Like Differential Expression (ALDEx2) was used to analyse differential abundance in ASVs between the groups [30]. A univariate test was performed to evaluate the differences in metabolic features and microbes between groups, where *p* < 0.05 was considered significant. A pairwise correlation analysis was performed using Spearman correlation to evaluate the correlations between microbes and metabolic indices, gene expression, and SCFAs, whereby R > 0.3 and *p* < 0.05 were considered significant.

### 2.12. Statistical Analysis

Data were expressed as mean ± SEM (standard error of mean). One way analysis of variance (ANOVA) with Tukey posthoc was used to compare the means among the groups following a normality test. A *p* value < 0.05 was considered statistically significant. All statistical analyses were performed using GraphPad Prism version 9.0 (GraphPad Software Inc., San Diego, CA, USA).

## 3. Results

### 3.1. Geraniin and GEE Characterisation

The total mass and purity of geraniin (90% purity) and GEE have been published elsewhere [25]. The LCMS analysis of GEE detected 26 compounds, including geraniin, chebulagic acid, and calenduloside H methyl ester (Supplementary Table S3). The identity of geraniin crystals was also confirmed with LCMS (Supplementary Figure S1). The minor impurities detected together with geraniin crystals were chebulagic acid and an unknown compound with a mass of 972.1077 (Supplementary Table S4).

### 3.2. Impact of Geraniin and GEE on Lipid Profile, Body Weight, Adiposity, and Energy Intake

**Figure 1:**
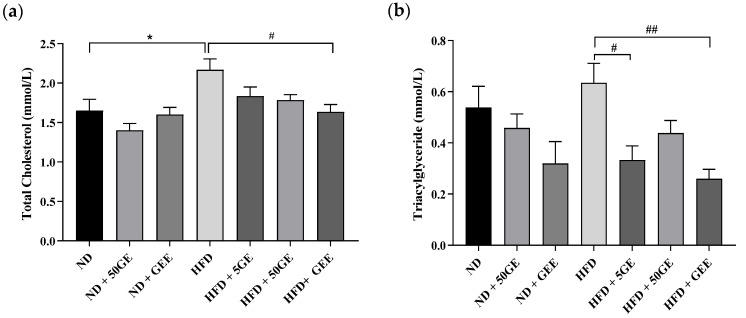

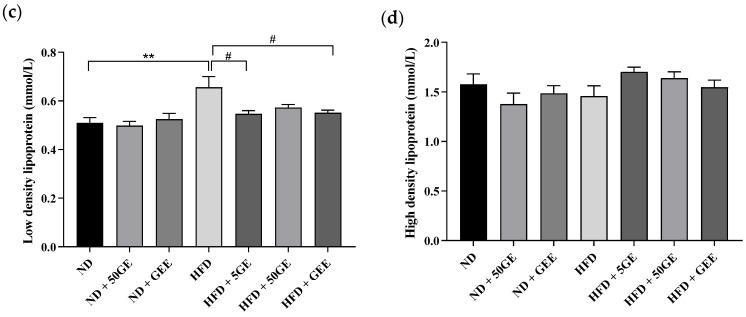
Effect of geraniin and GEE on lipid profile among the ND and HFD-fed rats. (**a**) Total cholesterol (TC), (**b**) triacylglyceride (TG), (**c**) low-density lipoprotein (LDL), (**d**) high-density lipoprotein (HDL). Sample size, *n* = 6. * *p* < 0.05, ** *p* < 0.005 HFD compared to ND. # *p* < 0.05, ## *p* < 0.005 HFD compared to the treatment groups fed an HFD.

The significant increase in total cholesterol (TC), low-density lipoprotein (LDL), and a non-significant increase in triacylglyceride (TG) with HFD consumption was attenuated by GEE (Figure 1a–c). An amount of 5 mg geraniin significantly lowered TG and LDL (Figure 1b,c) in the same group. In ND groups, GEE caused a noticeable reduction in TG (not significant), while other parameters were unaffected by both test compounds (Figure 1a–d).

HFD-induced significant weight gain and visceral adipose tissue (VAT) were reduced by 5 mg geraniin and GEE (non-significant) (Supplementary Figure S2). Geraniin and GEE treatment did not affect energy intake in ND and HFD groups (Supplementary Figure S2).

### 3.3. Impact of Geraniin and GEE on Glucose Homeostasis

**Figure 2:**
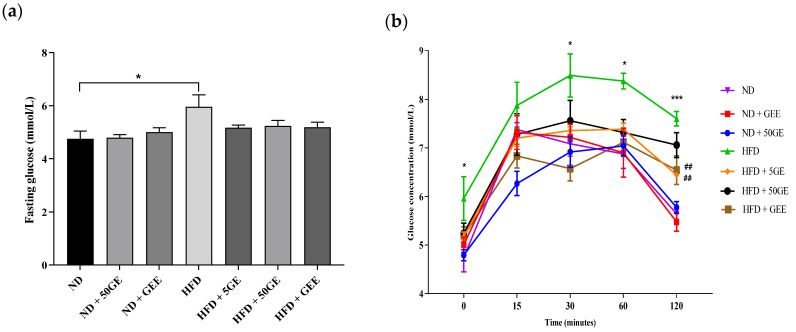

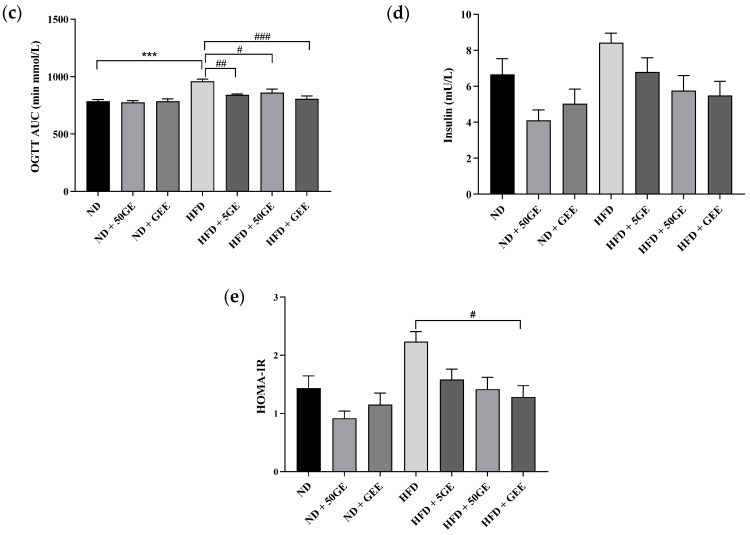
Effect of geraniin and GEE on glucose homeostasis among ND and HFD-fed rats. (**a**) Fasting blood glucose (FBG), (**b**) oral glucose tolerance test (OGTT), (**c**) OGTT AUC, (**d**) fasting insulin (FI), (**e**) homeostatic model assessment of insulin resistance (HOMA-IR). Sample size, *n* = 6. * *p* < 0.05, *** *p* < 0.0001 HFD compared to ND. # *p* < 0.05, ## *p* < 0.005, ### *p* < 0.0005 HFD compared to the treatment groups fed an HFD.

HFD intake alone significantly increased fasting blood glucose (FBG) (Figure 2a) and impaired glucose tolerance (Figure 2b,c), while the administration of geraniin and GEE among HFD-fed groups significantly improved glucose intolerance (Figure 2b,c). Interestingly, only GEE treatment significantly reduced HOMA IR (Figure 2e). Although not significant, FBG and fasting insulin (FI) were reduced in all HFD-treated groups (Figure 2a,d). Similarly, among ND-treated groups, a non-significant reduction in FI and HOMA IR were observed (Figure 2d,e).

### 3.4. Influence of Geraniin and GEE on Metabolic Endotoxemia, Gene Expression, and Plasma Cytokines

**Figure 3:**
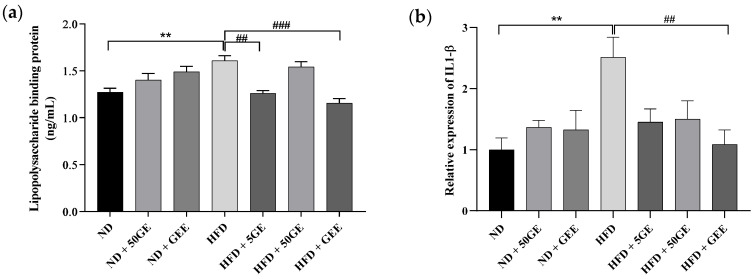

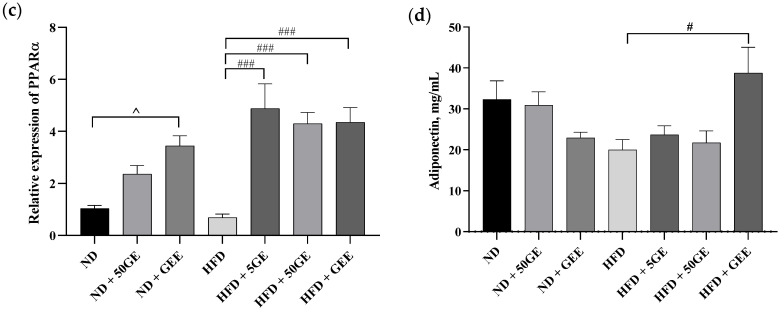
Effect of geraniin and GEE on metabolic endotoxemia, gene expression, and plasma cytokines in ND and HFD-fed rats. (**a**) Lipopolysaccharide-binding protein (LBP), (**b**) IL 1β, (**c**) PPARα, (**d**) adiponectin. Sample size, *n* = 4 to 8. ** *p* < 0.005 HFD compared to ND. # *p* < 0.05, ## *p* < 0.005, ### *p* < 0.0001 HFD compared to the treatment groups fed an HFD. ^ *p* < 0.05 ND + GEE compared to ND.

An amount of 5 mg geraniin and GEE supplementation were able to significantly reduce lipopolysaccharide-binding protein (LBP) elevation induced by HFD intake. Among the ND groups, no significant observation was noted (Figure 3a).

HFD consumption is associated with low-grade systemic inflammation and dysregulation of lipid metabolism, thus we investigated the effect of geraniin and GEE on the expression of inflammatory (TNFα, IL 6, IL 1 β) and lipogenic genes in the liver (PPARα) and rWAT (PPARγ). GEE attenuated the hepatic expression of IL 1β, which was significantly induced by HFD intake (Figure 3b). The other inflammatory genes (TNFα, IL 6) (Supplementary Figure S3) and PPARα were unaffected by HFD (Figure 3c). Nevertheless, all the treatment groups significantly upregulated hepatic PPARα expression (Figure 3c). Among the ND groups, GEE significantly increased expression of PPARα (Figure 3c), while the inflammatory genes were not affected in either of the treatment groups (Figure 3b, Supplementary Figure S3). Conversely, in rWAT, none of the inflammatory genes or PPARγ were significantly different between groups (Supplementary Figure S4).

Among the plasma cytokines measured (adiponectin, leptin, monocyte chemoattractant protein 1(MCP1), tumour necrosis factor alpha (TNFα), interleukin 6 (IL 6), interleukin 1β (IL 1β)), TNFα, IL 6, and IL 1β were undetected. Although HFD did not affect the cytokines tested, interestingly, only GEE supplementation significantly increased adiponectin (Figure 3d). Geraniin and GEE did not affect leptin and MCP1 in HFD groups (Supplementary Figure S3), while none of the ND treated groups exhibited a significant difference in the cytokines measured (Figure 3a,b and Supplementary Figure S3).

### 3.5. Influence of Geraniin and GEE on Plasma Short-Chain Fatty Acid Production

**Figure 4:**
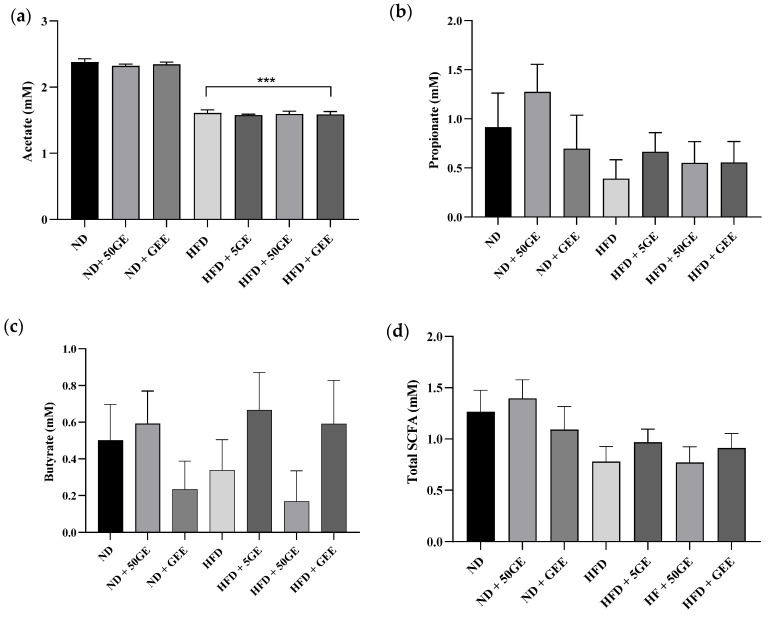
Effect of geraniin and GEE on short-chain fatty acid (SCFA) concentration in plasma among the ND and HFD-fed rats. (**a**) Acetate, (**b**) propionate, (**c**) butyrate, (**d**) total SCFA. Sample size, *n* = 8. *** *p* < 0.0001 HFD compared to ND.

HFD significantly reduced the concentrations of plasma acetate, and no significant difference was observed between the treated groups (Figure 4a). Concentrations of propionate and butyrate (Figure 4b,c) were not changed in the HFD group; however, an increasing trend, particularly for butyrate, was observed with 5 mg geraniin and GEE treatment (Figure 4c).

### 3.6. Effect of Geraniin and GEE on Gut Microbiota Composition

**Figure 5:**
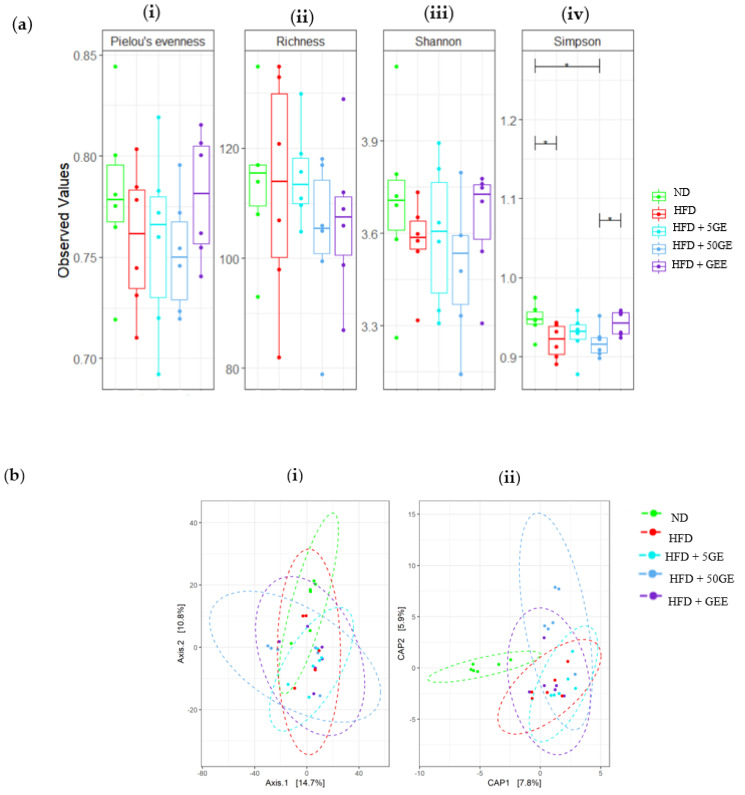

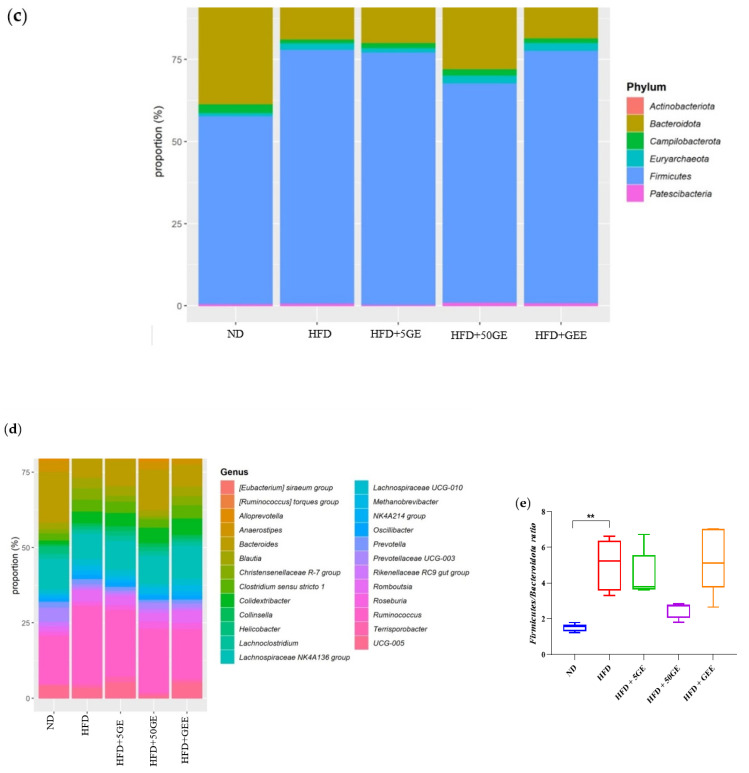
(**a**) Alpha diversity estimates based on (**i**) Pielou’s evenness, (**ii**) richness index, (**iii**) Shannon index, (**iv**) Simpson index. (**b**) Beta diversity based on (**i**) principal coordinate analysis (PCoA), (**ii**) canonical analysis of principal coordinates (CAP). The relative abundance of bacteria: (**c**) phyla, (**d**) genera, (**e**) Firmicutes/Bacteroidota ratio (F/B ratio) among ND, HFD, HFD5GE, HGD50GE, and HFDGEE. Sample size, *n* = 6, * *p* < 0.05, ** *p* < 0.005.

Alpha diversity was presented as Pieolou’s evenness, richness index, Shannon index, and Simpson index (Figure 5a(i–iv)). The Simpson index was significantly lower in HFD compared to the ND group, while no significance was noted between the HFD and treatment groups. None of the other indices were significantly different between the groups.

In this study, the beta diversity was evaluated using Aitchison distance-based principal coordinate analysis (PCoA), canonical analysis of principal coordinates (CAP) (Figure 5b(i,ii)), and PERMANOVA. The separation between the HFD and ND was apparent in the “constrained” CAP plot but not in the “unconstrained” PCoA plot. Overall, supplementation of geraniin and GEE did not promote structural changes in bacterial composition, except between the GEE and HFD (P. adjusted sig = 0.035) groups.

The effects of geraniin and GEE on the relative abundance of bacterial phyla (Figure 5c), genera (Figure 5d), and Firmicutes/Bacteroidota ratio (F/B ratio) (Figure 5e) were assessed in ND, HFD, and HFD treated rats. Firmicutes and Bacteroidota account for >95% of the phyla detected in the faecal samples of all groups. The ND group had a significantly higher abundance of Bacteroidota (*p* < 0.1) and Compilobacterota (*p* < 0.1) compared to the HFD group (Supplementary Figure S5). In comparison with HFD, a non-significant reduction in the relative abundance of Firmicutes and an increase in Bacteroidota were observed in all the treatment groups. The F/B ratio was significantly higher in the HFD group compared to ND (*p* < 0.05), which was non-significantly reduced by geraniin. Although there was a non-significant improvement in gut microbiota composition following treatment at the genus level, supplementation of geraniin and GEE increased the relative abundance of a few butyrate-producing bacteria. The butyrate producers identified were *Alloprevotella* (5 mg geraniin, 50 mg geraniin, GEE), *Blautia* (5mg geraniin, GEE), *Eubacterium siraeum* group (50 mg geraniin, GEE), *Lachnospiraceae NK4A136* group (GEE), *Clostridium sensu stricto* 1 (GEE), and *Ruminococcus torques* group (GEE).

### 3.7. Correlation between Gut Microbiota and Metabolic Parameters and Biomarkers

Pairwise correlations were conducted to identify specific correlations between various genera and metabolic parameters, inflammatory markers, gene expression, and SCFA between HFD and treatment groups (Table 1). Treatment with 5 mg geraniin saw *Clostridium sensu stricto 13* exhibiting a positive correlation with IL 6 (rWAT) and a negative correlation with HOMA IR and FI, while *Candidatues Saccharimonas* displayed a negative correlation with IL 6 (rWAT). Administration of 50 mg geraniin saw *Christensenellaceae R7 and the NK4A214* group showing positive correlations with FI and negative correlations with PPARα and IL 6 (rWAT). Supplementation with GEE saw *Clostridia UCG 014* presenting positive correlations with leptin and TG, while *Ruminococcus* presented positive correlations with leptin, TG, and FI.

## 4. Discussion

In the present study, we investigated the effect of geraniin (5 mg and 50 mg) and 115 mg GEE (equivalent geraniin concentration of 50 mg GE) on gut microbiota composition, metabolic endotoxemia, cytokines, gene expression (inflammation and lipid metabolism), short-chain fatty acids (SCFAs), and metabolic parameters in an HFD-induced metabolic syndrome animal model. We demonstrated that the consumption of HFD caused weight gain, hyperglycaemia, dyslipidaemia, metabolic endotoxemia, and gut microbiota alteration, which were ameliorated by 5 mg geraniin and 115 mg GEE. To the best of our knowledge, this is the first ever study to elucidate the effect of geraniin and GEE on gut microbiota composition and SCFAs. Our findings indicate the possibility of developing mainly GEE as a nutraceutical and potentially a prebiotic in managing diet-induced metabolic syndrome.

The supplementation of geraniin and GEE in the group fed on a normal diet did not significantly affect most metabolic parameters. However, a marginal reduction in visceral adipose tissue (VAT), fasting insulin (FI), and homeostatic model assessment of insulin resistance (HOMA IR) was noted. Studies have shown that normal-weight individuals with high body fat tend to be insulin resistant [31]. As such, supplementation of plant-based compounds such as geraniin or GEE may be beneficial in lowering the adiposity and subsequently insulin resistance among such individuals.

The antilipidemic property of geraniin was reported previously using various doses of geraniin [20,21,22]. However, none have reported on GEE. Geraniin at 25 mg/kg was observed to modulate hepatic lipid metabolic pathways such as synthesis of coenzyme A, fatty acid, and cholesterol metabolism in SD rats [22]. In line with this, we also observed a significant upregulation of hepatic PPARα in all the treatment groups. PPARα regulates genes involved in mitochondrial and peroxisomal fatty acid oxidation, which reduces TG accretion in circulation [32].

The antilipidemic effect observed in this study and inhibition of α-amylase reported previously [19] explain the significant improvement in OGTT by geraniin and GEE. TG overload in the pancreas has been demonstrated to disrupt β-cell function [33] and induce oxidative stress (OS) [34]. Hence, reduction in TG appears to have also improved glucose tolerance. Alpha-amylase metabolises carbohydrates into glucose. Consequently, inhibition of this enzyme reduces plasma glucose [19].

Interestingly, only GEE significantly improved HOMA IR with a simultaneous significant increase in adiponectin concentration. Adiponectin was observed to attenuate insulin resistance by restoring the antioxidant system in an HFD animal model [35]. We also believe that the induction of adiponectin is related to the metabolites of GEE since incubation of geraniin and GEE with 3T3 L1 adipocytes did not show changes in adiponectin level [36].

This study did not observe upregulation of proinflammatory cytokines as previously reported [37], despite a significant increase in systemic LBP level. This is congruent with the hepatic (TNF, IL 6) and retroperitoneal white adipose tissue (TNFα, IL 1β, IL 6) inflammatory gene expressions. This may be because inflammatory responses are recognised as rapid, transient, and highly variable between animals, and can only be detected at specific intervals [38]. Therefore, the propensity to develop systemic inflammation was suggested to be influenced by the genetic makeup of the experimental animal, dietary composition, and study duration [38,39].

The alpha diversity indices were not significantly affected by geraniin and GEE, indicating that the richness and evenness of gut microbiota are very similar across groups. A significant difference in beta diversity between GEE and HFD was observed; however, this was not apparent in PCoA and CAP plots. Similar observations were recorded with quercetin [40], despite significant differences between taxa. Ellagitannin-containing extracts such as jamun [14] and pomegranate peel [13] have reported a significant alteration in phyla and genus. However, we did not observe such changes. This could be related to the duration of interventions, where test compounds were administered between 8 and 16 weeks in these studies. Moreover, test compounds were administered simultaneously with HFD (prevention model), whereas in our study, the test compounds were administered following eight weeks of HFD intake (treatment model). Most notably, the hypervariable regions and bioinformatic tools utilised also differed from ours, which may have influenced the outcome of this study.

Ellagitannin undergoes hydrolysis to release ellagic acid (EA) upon consumption, which is then converted by gut microbiota (mainly in the colon) to various urolithins [13]. In HFD animal models, EA and urolithins were demonstrated to attenuate HFD-induced OS, hence the metabolic derangements [41,42]. Therefore, we speculate that the improvement in metabolic aberrations observed in this study is likely due to the metabolites of geraniin and GEE. This is because the breakdown of ellagitannin has been reported to precede significant alteration on gut microbiota composition [43]. Hence, integration of metabolomic analysis to detect geraniin and GEE metabolites is warranted in future studies.

Despite not achieving statistical significance, we noticed a positive modulation in several butyrate-producing bacteria in all the treatment groups. Accordingly, the correlation analysis also supported significant associations between butyrate producers and various metabolic markers and inflammatory genes. This implies that butyrate may have played a role in promoting metabolic health. Interestingly, a higher plasma butyrate concentration was mainly detected in 5 mg geraniin and 115 mg GEE. Butyrate, an antioxidant, is an energy source for the enterocytes and plays a pivotal role in improving gut integrity [44]. In HFD studies, administration of butyrate was proven to ameliorate metabolic aberrations [44]. Similarly, we also observed a significant modulation in TG, LDL, OGTT, HOMA IR, and LBP with 5 mg geraniin and GEE supplementation.

An amount of 5 mg geraniin was more effective than 50 mg geraniin as TG, LDL, and LBP were significantly attenuated at this dose. A similar observation was noted by Phang et al. in 2019 [21], whereby 3.13 mg/kg geraniin exerted significant changes in metabolic parameters compared to higher doses. Furthermore, in 2006 Whitley et al. [45] showed that high-concentration EA accumulates in caco 2 cells, resulting in its reduced transcellular transport. Thus, we postulate that more EA may have accumulated in the intestinal epithelium at a higher concentration of geraniin, interfering with the transportation of gut-derived metabolites into the systemic circulation. In line with this, we observed a lower concentration of butyrate following 50 mg geraniin supplementation.

We also noted that the 115 mg GEE that corresponds to 50 mg geraniin was more effective than geraniin in improving metabolic disturbances as it significantly reduced TC, TG, LDL, HOMA IR, and LBP. This outcome could be related to the synergetic effect of other compounds in the crude extract. The LCMS analysis of the crude extract used in our study revealed 26 compounds in the extract. These compounds may have led to the production of other biologically active metabolites. Additionally, the presence of other compounds may have affected the concentration of gut-derived metabolites. Accordingly, we also observed a noticeable increase in butyrate production following GEE supplementation.

### Study limitations

Although our study provided evidence of geraniin and GEE’s beneficial effects, some limitations need to be acknowledged. GCMS is more sensitive in quantifying SCFA compared to HPLC. Apart from that, metabolomic profiling of EA and urolithins in biological samples will help understand the role of gut microbiota in ameliorating metabolic parameters. Additionally, assessment of gut microbiota before treatment would have allowed for the intragroup comparison in gut microbiota following treatment. We also believe a longer intervention period is vital for significant alteration in the gut microbial composition.

## 5. Conclusions

In conclusion, our findings demonstrate that the administration of 5 mg/kg geraniin and 115 mg/kg geraniin-enriched extract significantly improved triacylglyceride, low-density lipoprotein, glucose intolerance, adiponectin, HOMA IR, and metabolic endotoxemia. It appears that the protective role exerted by these compounds was related to the butyrate-producing bacteria, increased synthesis of butyrate, and the upregulation of PPARα. Moreover, our study showed that 115 mg/kg geraniin-enriched extract was more effective compared to 5 mg/kg and 50 mg/kg geraniin in ameliorating HFD-induced metabolic aberrations. This is probably related to other compounds in the extract and the concentration of short-chain fatty acids (particularly butyrate). Nevertheless, long-term studies investigating changes in gut microbial strains and metabolomics to detect EA and urolithins may help elucidate the ameliorative role of this ellagitannin against diet-induced metabolic syndrome.

## Figures and Tables

**Table 1:** Pairwise correlations between gut microbiota and metabolic markers, IL 6 gene, PPARα gene.

Groups	Genus	Metabolic Marker/Gene	Correlation, r	*p* Value
HFD vs. HFD + 5 GE	*Clostridium sensu stricto 13*	HOMA IR	−0.76	4.10 × 10^−3^
		FI	−0.743	5.70 × 10^−3^
		IL 6 gene (rWAT)	0.583	0.046
	*Candidatus Saccharimonas*	IL 6 gene (rWAT)	−0.633	0.027
HFD vs. HFD + 50 GE	*Christensenellaceae R 7 group*	PPARα gene (L)	−0.651	0.022
		FI	0.581	0.047
		IL 6 gene (rWAT)	−0.639	0.025
	*NK4A214 group*	PPARα gene (L)	−0.613	0.034
		FI	0.62	0.032
		IL 6 gene (rWAT)	−0.674	0.016
HFD vs. HFD + GEE	*Clostridia UCG 014*	Leptin	0.636	0.026
		TG	0.727	7.40 × 10^−3^
	*Ruminococcus*	FI	0.685	0.014
		Leptin	0.594	0.042
		TG	0.615	0.033

Homeostatic model assessment of insulin resistance (HOMA IR), geraniin (GE), geraniin-enriched extract (GEE), fasting insulin (FI), interleukin 6 (IL 6), retroperitoneal white adipose tissues (rWAT), peroxisome proliferator-activated receptor alpha (PPARα), triacylglyceride (TG).

## Data Availability

Data are contained within the article.

## Supplementary Materials

The following supporting information can be downloaded at https://www.mdpi.com/article/10.3390/antiox11040632/s1. Table S1: Composition of macronutrients and ingredients of AIN93G diet; Table S2: Accession numbers, and forward and reverse primers of the reference and target genes; Table S3: List of compounds in GEE; Figure S1: Identification of geraniin using LCMS; Table S4: List of compounds in GE; Figure S2: Body weight, adiposity, and energy intake; Figure S3: Plasma cytokines and hepatic inflammatory genes; Figure S4: The relative expression of inflammatory genes and PPARγ in rWAT; Figure S5: MA and effect plots to determine the significance of phyla between groups.
