# Supplementary material for: The Prebiotic Potential of Geraniin and Geraniin-Enriched Extract against High-Fat-Diet-Induced Metabolic Syndrome in Sprague Dawley Rats
Source: Antioxidants (Basel). 2022 Mar 25;11(4):632. doi: 10.3390/antiox11040632 (PMC9029067; doi:10.3390/antiox11040632)
Supplement: Supplementary file 1 [file antioxidants-11-00632-s001.zip › antioxidants-1622703-supplementary.pdf]

Supplementary Materials

Table S1. Composition of macronutrient and ingredients of AIN93G diet.

Macronutrient	Normal diet (D10012GR)		High-fat diet (D12033001)	
	Mass, g%	kcal, %	Mass, g%	kcal, %
Protein	20	20	27	20
Carbohydrate	64	64	27	20
Fat	7	16	35	60
**Total**		**100**		**100**
**Kcal/gm**	**4.0**		**5.3**	
**Ingredient**	**Mass, g**	**kcal**	**Mass, g**	**kcal**
Casein	200	800	200	800
L-Cystine	3	12	3	12
Corn starch	397.486	1590	0	0
Maltodextrin 10	132	528	123.8	495
Sucrose	100	400	68	272
Cellulose, BW200	50	0	50	0
Soybean oil	70	630	25	225
Lard	0	0	239.5	2156
t-Butylhydroquinone	0.014	0	0	0
Mineral Mix S10022G	35	0	35	0
Vitamin Mix V10037	10	40	10	40
Choline Bitartrate	2.5	0	2.5	0
**Total**	**1000.05**	**4000**	**756.8**	**4000**

Table S2. Accession numbers, forward and reverse primers of the reference and target genes.

Target and reference genes	Accession Number	Primer sequence
Tumour Necrosis Factor alpha (Tnfα)	NM_012675	5′-GTCTTTGAGATCCATGCCATTG-3′<br>5′-AGACCCTCACACTCAGATCA-3′
Interleukin 6 (IL-6)	NM_012589	5′-CCTTCTGTGACTCTAACTTCTCC-3′<br>5-CAGAGCAATACTGAAACCCTAGT-3′
Interleukin 1 beta (IL-1β)	NM_031512	5′-TTGTCGTTGCTTGTCTCTCC-3′<br>5-GTGCTGTCTGACCCATGT-3′
Peroxisome proliferator-activated receptor gamma (PPARγ)	NM_001145366	5′-GGACGCTGAAGAAGAGACCTG-3′<br>5′-CCGGGTCCTGTCTGAGTATG-3′
Peroxisome proliferator-activated receptor alpha (PPARα)	NM_013196	5′-TCACACAATGCAATCCGTTT-3′<br>5′-GGCCTTGACCTTGTTCATGT-3′
Glyceraldehyde 3-phosphate dehydrogenase (GAPDH)	NM_017008	5′-TCACCACCATGGAGAAGGC-3′<br>5′-GCTAAGCAGTTGGTGGTGCA-3′
Lactate dehydrogenase (LDHA)	NM_017025	5'-GTTGTTGGGGTTGGTGCTGTT-3′<br>5′-CCACTGGGTTTGAGACGATGA-3′

Table S3. List of compounds in GEE.

m/z	Formula	Label	Mass	Base Peak	RT
554.0714		Cpd 5: 4.372	1110.1574	554.0714	4.372
577.1618	C33 H26 N2 O8	Cpd 4: C33 H26 N2 O8	578.1693	577.1618	4.305
633.0966	C27 H24 N O17	Cpd 2: C27 H24 N O17	634.1039	633.0966	4.228
649.4176	C32 H56 N7 O7	Cpd 24: C32 H56 N7 O7	650.4248	649.4176	22.88
666.4066	C30 H49 N15 O3	Cpd 25: C30 H49 N15 O3	667.4137	666.4066	22.881
781.4515		Cpd 12: 21.668	782.4588	781.4515	21.668
785.1016	C37 H30 N4 O10 S3	Cpd 7: CI Acid red 114	786.1093	785.1016	4.997
795.4643		Cpd 11: 21.582	796.4716	795.4643	21.582
798.4412	C38 H69 N O14	Cpd 13: 14-Hydroxy-6-O-methylerythromycin A	763.4719	798.4412	21.668
812.453		Cpd 10: 21.582	813.4603	812.453	21.582
823.4569		Cpd 26: 23.635	824.4642	823.4569	23.635
923.5029	C45 H82 O15 P2	Cpd 9: CL(1'-[18:2(9Z,12Z)/0:0],3'-[18:2(9Z,12Z)/0:0])	924.5099	923.5029	21.511
923.5031	C45 H82 O15 P2	Cpd 21: CL(1'-[18:2(9Z,12Z)/0:0],3'-[18:2(9Z,12Z)/0:0])	924.5102	923.5031	22.479
923.5046	C45 H82 O15 P2	Cpd 17: CL(1'-[18:2(9Z,12Z)/0:0],3'-[18:2(9Z,12Z)/0:0])	924.5116	923.5046	21.818
951.0773	C41 H28 O27	Cpd 3: Geraniin	952.0848	951.0773	4.254
953.0908	C41 H30 O27	Cpd 8: Chebulagic acid	954.0972	953.0908	5.04
959.4779	C45 H82 O15 P2	Cpd 15: CL(1'-[18:2(9Z,12Z)/0:0],3'-[18:2(9Z,12Z)/0:0])	924.508	959.4779	21.812
959.4779	C45 H82 O15 P2	Cpd 18: CL(1'-[18:2(9Z,12Z)/0:0],3'-[18:2(9Z,12Z)/0:0])	924.5081	959.4779	22.031
969.505	C49 H78 O19	Cpd 22: Calenduloside H methyl ester	970.512	969.505	22.479
969.5072	C49 H78 O19	Cpd 14: Calenduloside H methyl ester	970.514	969.5072	21.812
969.5079	C49 H78 O19	Cpd 19: Calenduloside H methyl ester	970.5147	969.5079	22.033
971.1004		Cpd 1: 3.856	972.1077	971.1004	3.856
986.4944		Cpd 23: 22.480	987.5016	986.4944	22.48
986.4953		Cpd 16: 21.813	987.5025	986.4953	21.813
986.4958		Cpd 20: 22.034	987.5031	986.4958	22.034
1011.5121		Cpd 27: 24.105	1012.5193	1011.5121	24.105
1028.4997		Cpd 28: 24.106	1029.507	1028.4997	24.106
1109.0846		Cpd 6: 4.375	1110.0918	1109.0846	4.375

Mass-to-charge ratio (m/z), retention time (RT).

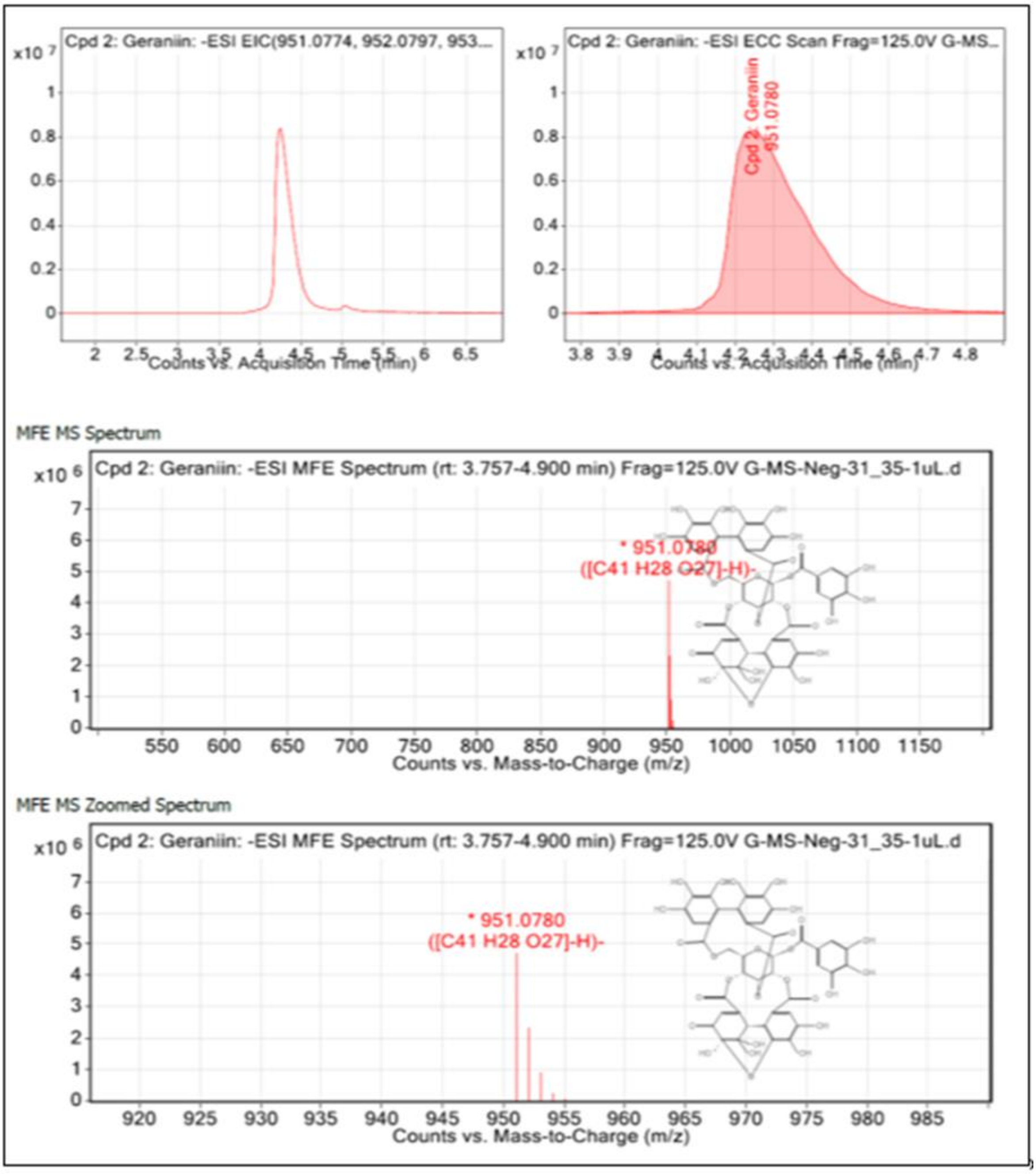

Figure S1. Identification of geraniin using LCMS. Mass spectrum shows molecular ion for geraniin $C_{41}H_{27}0_{27}^{-}$ which was detected at negative mode with m/z 951.0780

Table S4. List of compounds in GE.

m/z	Formula	Label	Mass	Base Peak	RT
951.078	C41 H28 O27	Cpd 2: Geraniin	952.0852	951.078	4.272
953.09	C41 H30 O27	Cpd 3: Chebulagic acid	954.0965	953.0899	5.049
971.1004		Cpd 1: 3.853	972.1077	971.1004	3.853

Mass-to-charge ratio (m/z), retention time (RT).

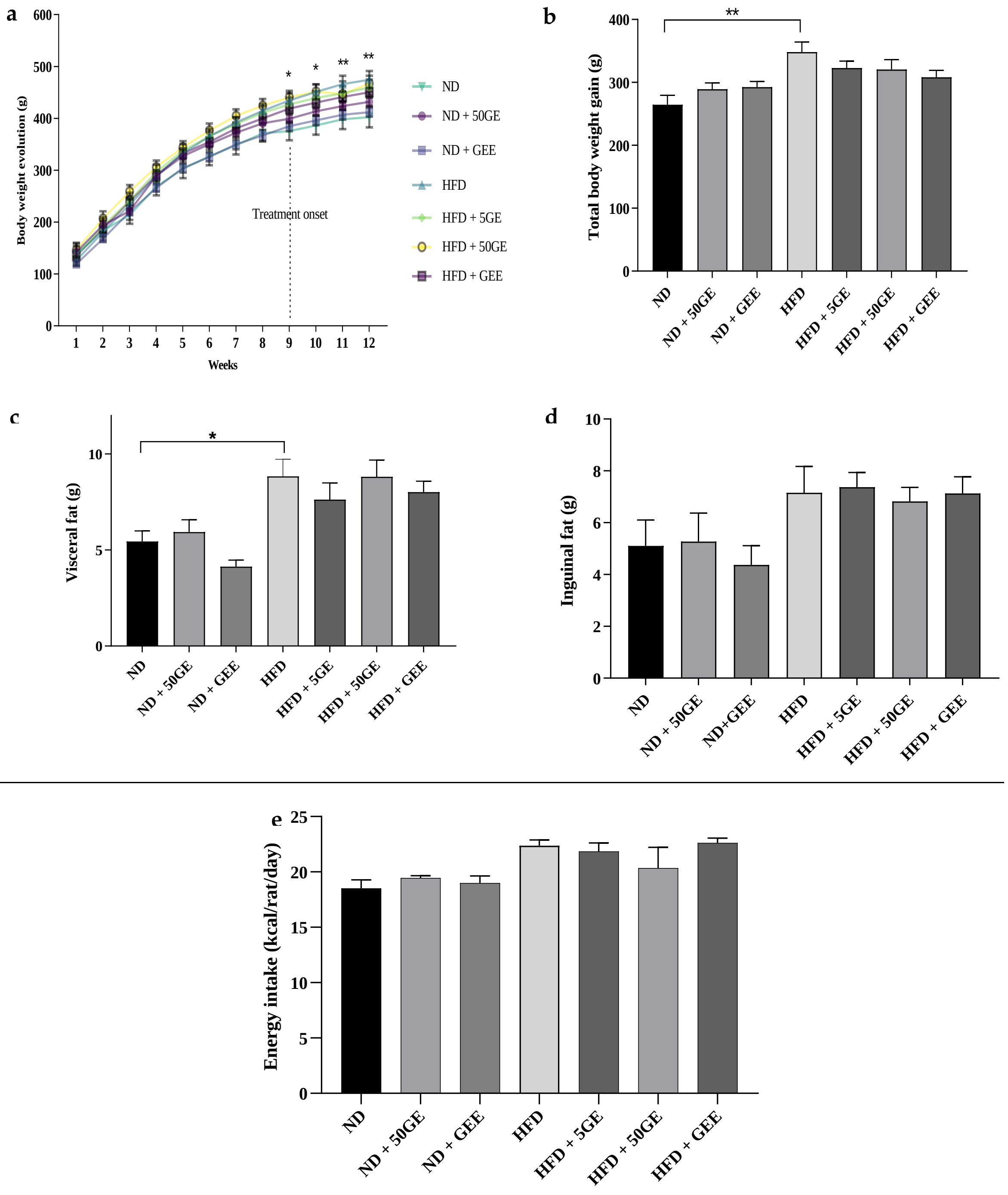

Figure S2. Body weight, adiposity and energy intake. Effect of geraniin and GEE in ND and HFD-fed rats. (a) Bodyweight evolution, (b) Total body weight gain, (c) Visceral adipose tissue (VAT), (d) Inguinal adipose tissue (IAT), (e) Energy intake. Sample size, n = 6. * $p < 0.05$, ** $p < 0.005$ HFD compared to ND.

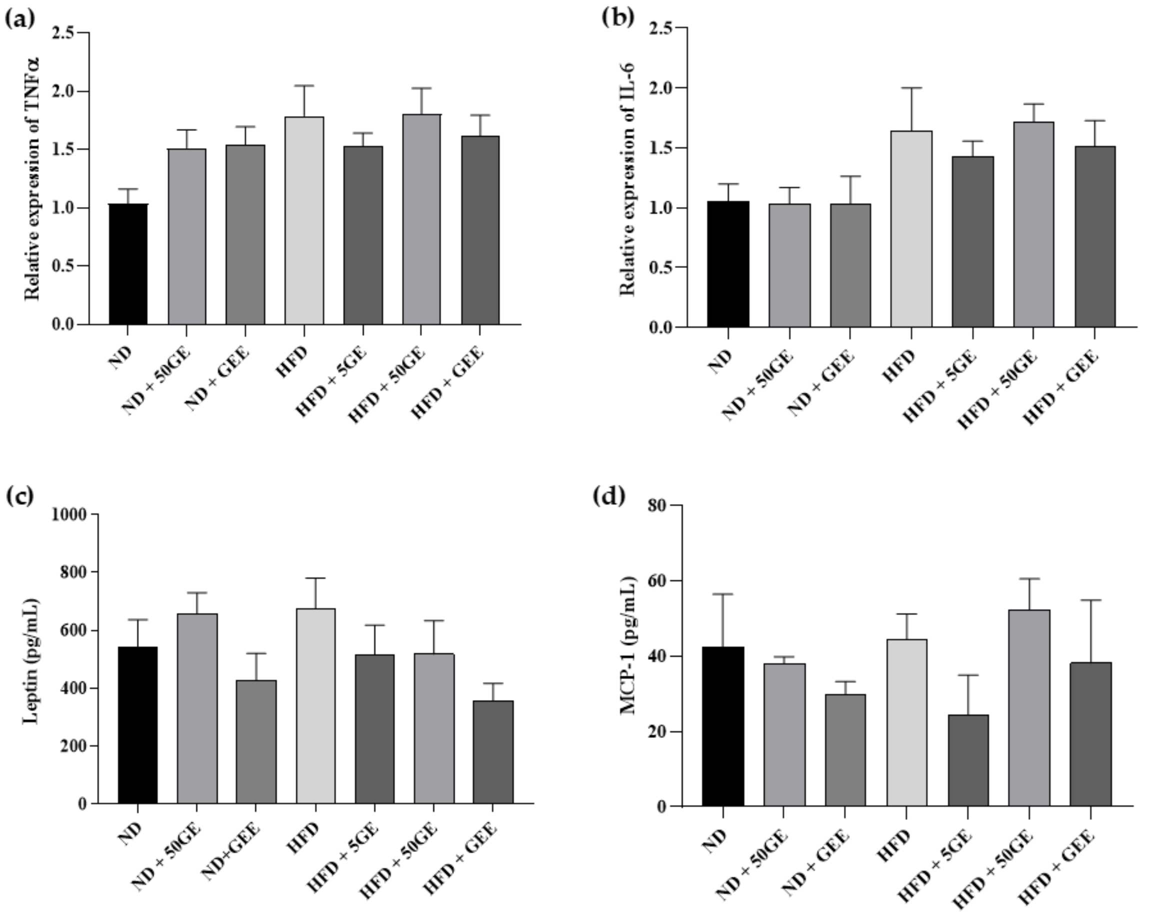

Figure S3. Plasma cytokines and hepatic inflammatory genes. Effect of geraniin and GEE on plasma cytokines and hepatic inflammatory genes in ND and HFD-fed rats. (a) (c) TNFα, (d) IL 6 (c) Leptin, (d) Monocyte chemo-attractant protein 1 (MCP 1). Sample size, n = 4 to 6

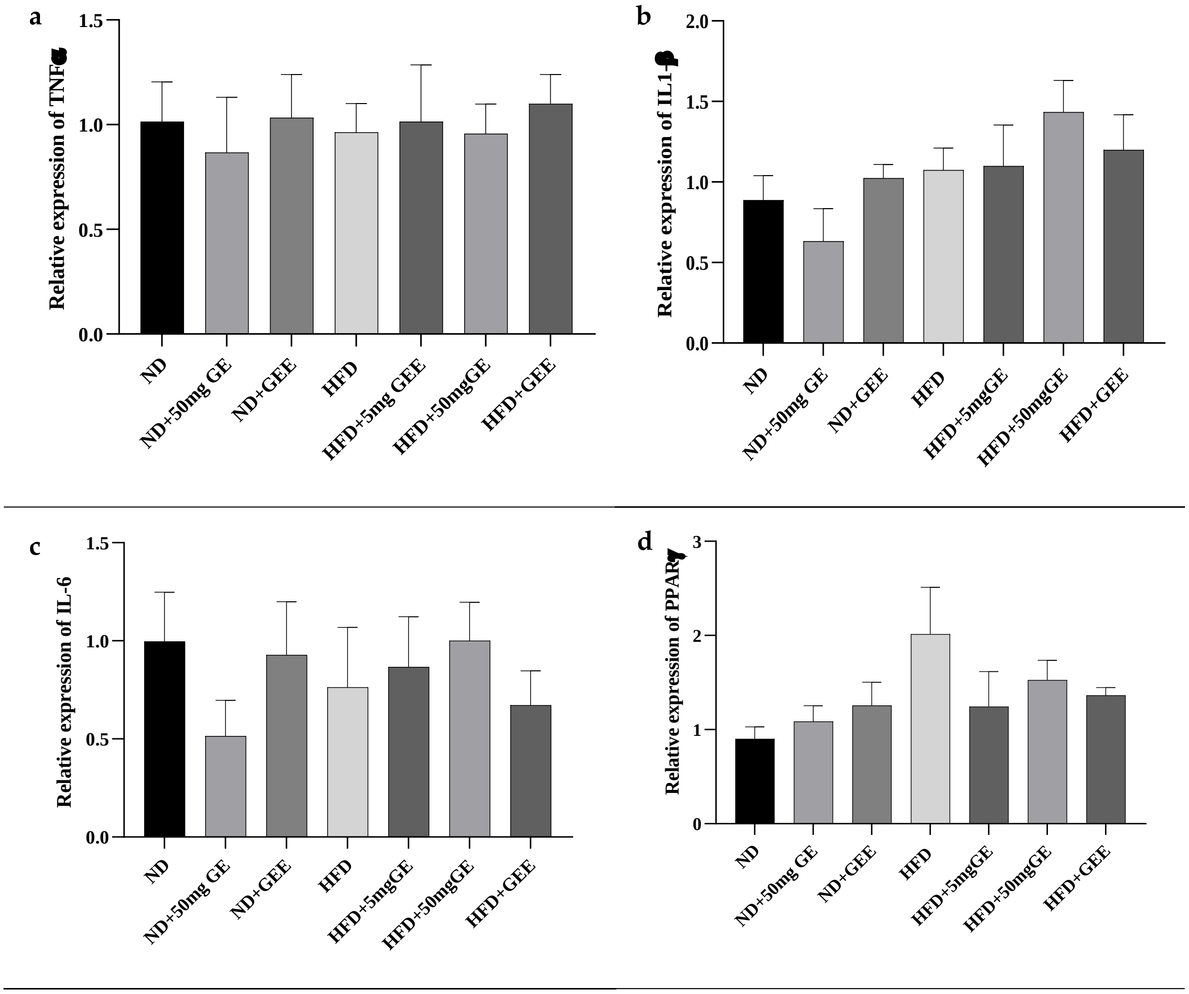

Figure S4. The relative expression of inflammatory genes and PPARγ in rWAT. Relative expression of inflammatory genes in rWAT (a) TNF*α* (b) IL-1β (c) IL-6, (d) PPARγ. Sample size, n = 5 to 8. No significant difference between groups

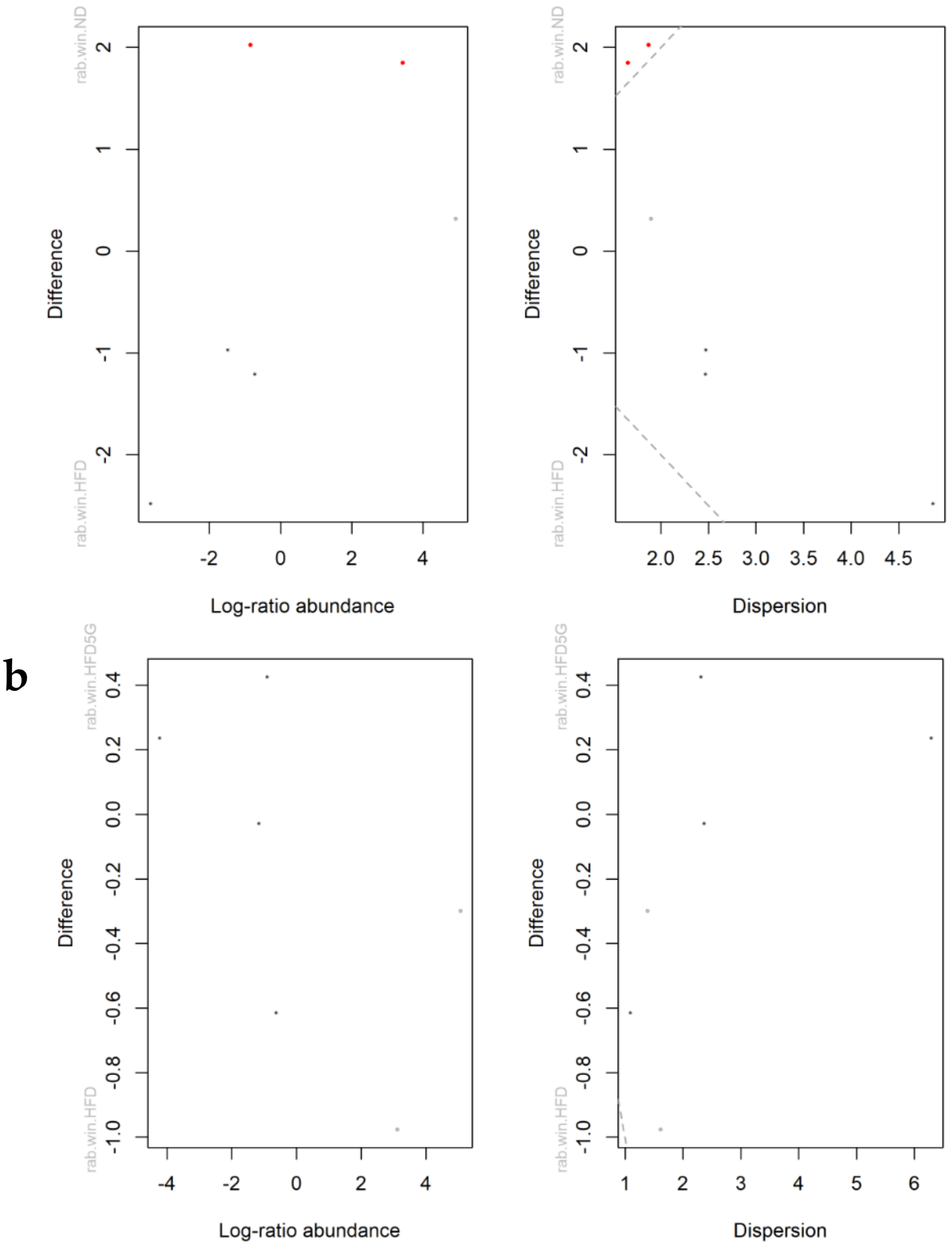
rab.win.ND
rab.win.HFD
Difference
Log-ratio abundance
Dispersion
b
rab.win.HFD5G
rab.win.HFD
Difference
Log-ratio abundance
Dispersion

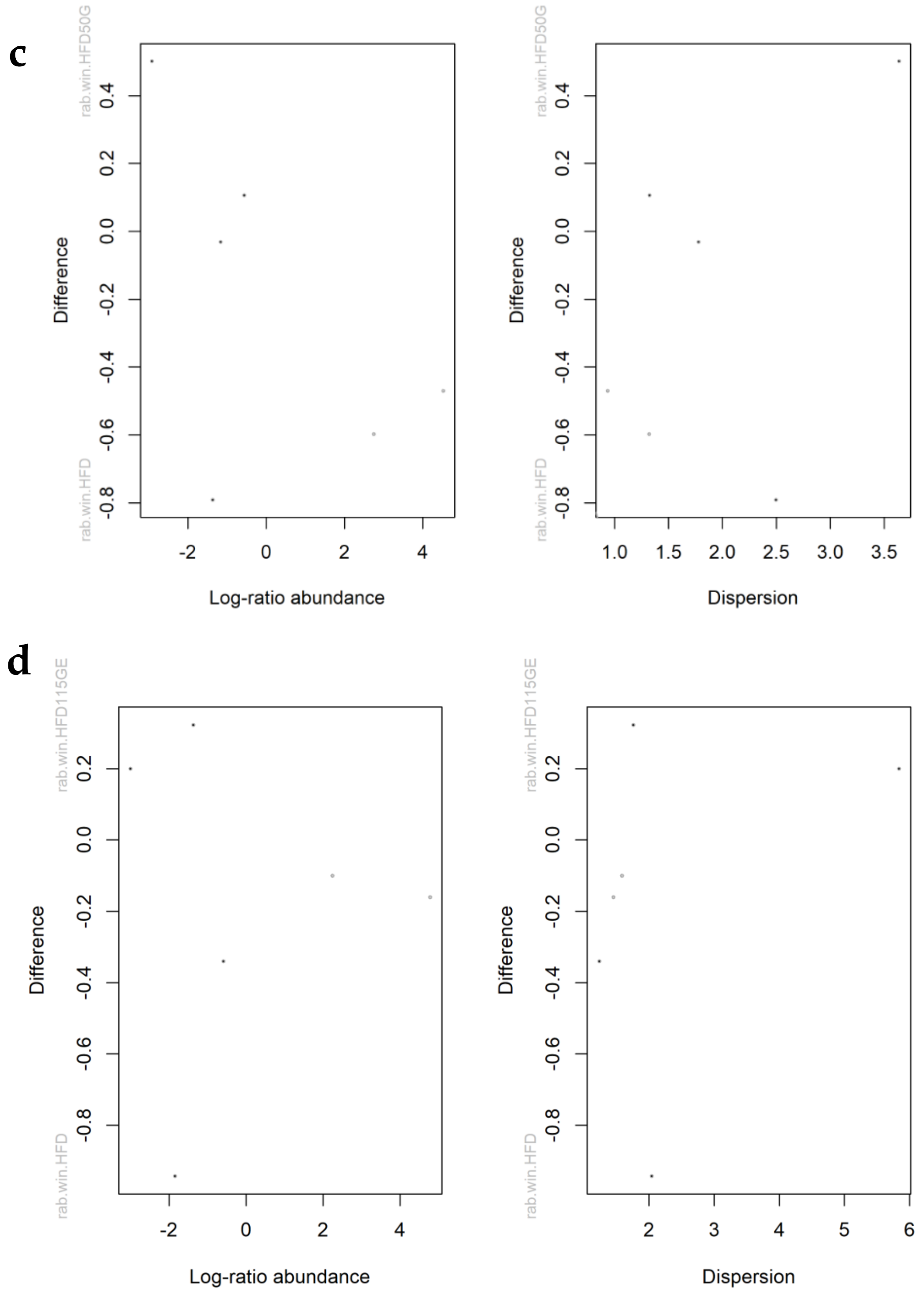

.

Figure S5. MA and effect plots to determine the significance of phyla between groups. MA and effect plots following ALDEx2 analysis to determine significance at phyla level between (a) HFD vs ND, (b) HFD vs HFD + 5GE, (c) HFD + 50GE, (d) HFD + 115GEE. A significant difference between HFD and ND was detected at the phyla level (red dots) following the Benjamini–Hochberg correction for false-positive rate
